# Draft Genome Sequences of 15 Multidrug-Resistant Escherichia coli Strains Isolated from Indigenous Foods and Food-Gathering Sites in Aotearoa, New Zealand

**DOI:** 10.1128/mra.01158-21

**Published:** 2022-04-26

**Authors:** Sophie van Hamelsveld, Gayle C. Ferguson, Brigitta Kurenbach, Deborah J. Paull, Irai Weepu, Jack A. Heinemann

**Affiliations:** a School of Biological Sciences, Te Whare Wānanga o Waitaha/University of Canterbury, Christchurch, New Zealand; b School of Natural and Computational Sciences, Massey University, Auckland, New Zealand; c Kaunihera Taiao ki Waitaha/Environment Canterbury, Christchurch, New Zealand; University of Arizona

## Abstract

We report the draft genomes of 15 multidrug-resistant and potentially pathogenic Escherichia coli strains isolated from watercress, cockles, or the surrounding water in Aotearoa, New Zealand.

## ANNOUNCEMENT

Information on antimicrobial resistance (AMR) in the New Zealand environment is sparse (1). Bivalve species such as tuaki (littleneck cockle [Austrovenus stuchburyii]) concentrate bacteria (2) and therefore may be sentinels of AMR in marine environments (3) or sources of food safety risk. Wātākirihi (watercress [Nasturtium officinale]), a freshwater vegetable, was shown to be a carrier of Escherichia coli (4). Food poisoning outbreaks overseas were attributed to watercress and shellfish (5, 6), while recreational water contact is a risk factor for pathogenic E. coli carriage in New Zealand (7). Therefore, we selected E. coli strains isolated in Waitaha/North Canterbury from watercress, cockles, and the surrounding water for whole-genome sequencing. Multidrug-resistant isolates were prioritized for sequencing.

Strains CK5_JAN2020, CK5_MAY2020, and CKCHL2_MAY2020 were isolated from cockles following New Zealand Ministry for Primary Industries guidelines (8). Strains CSCIP4_JAN2020 and CSCIP1_MAR2020 were isolated from watercress as follows. Watercress leaves (50 g) were blended for 2 min with 150 mL sterile phosphate-buffered saline. Aliquots (1 mL) were aseptically spread on tryptone-bile-X-glucuronide (TBX) agar. Other strains were isolated by membrane filtration from water collected at food-gathering sites (9). Plates were incubated for 12 to 16 h at 44°C before presumptive E. coli strains were saved for further analysis.

Methods for determining MIC values and conjugation conditions were as described previously (10, 11). DNA was extracted from single colonies cultured on TBX agar and was sequenced using Illumina paired-end sequencing at the Microbial Genome Sequencing Center (MiGS) (Pittsburgh, PA, USA). Raw reads were trimmed with Trimmomatic v0.39 (12) and assembled with SPAdes v3.15.4 (13). AMR genes (ARGs) were annotated with ResFinder v4.1 (14, 15), and multilocus sequence typing (MLST) was performed using MLST v2.0 (16). Public-facing genomes were annotated by PGAP v5.3 (17). Default parameters were used for all software. Sequencing metrics and strain data are presented in Table 1.

**TABLE 1:** Genome statistics and genotyping and phenotyping information

Strain	Sampling location^*a*^	No. of reads (million)	Amt of DNA sequenced (Mb)	Genome length (bp)	*N*_50_ (bp)	Coverage depth (×)	GC content (%)	No. of contigs	GenBank accession no.	BioSample accession no.	SRA accession no.	ST	AMR phenotype^*b*^	ARGs
CK5_JAN2020	1	1.6	473.3	4,927,028	133,402	96	50.6	98	JAJNOX000000000	SAMN23498528	SRR17163598	155	Tet, Tmp	*aadA5*, *dfrA17*, *sul1*, *tet*(A)
ASH.EST_CHL1_JAN2020	1	1.6	578.9	5,128,945	184,110	90	50.7	91	JAJNOY000000000	SAMN23498529	SRR17163597	654	Amp, Tet, Tmp, Chl	*aadA2*, *aph(3″)-Ib*, *aph(6)-Id*, *bla*_TEM-1B_, *dfrA12*, *floR*, *sul2*, *sul3*, *tet*(A)
CK5_MAY2020	1	1.5	537.7	5,026,556	182,991	86	50.7	72	JAJNOZ000000000	SAMN23498530	SRR17163596	1722	Amp, Tet, Tmp, Chl	*aadA1*, *aadA2*, *bla*_TEM-1B_, *cmlA1*, *dfrA12*, *sul3*, *tet*(A)
CKCHL2_MAY2020	1	1.5	559.3	4,847,565	110,687	92	50.8	91	JAJNPA000000000	SAMN23498531	SRR17163595	10	Amp, Tet, Tmp, Chl	*bla*_TEM-1B_, *dfrA14*, *sul2*, *tet*(A)
CAMP2_JAN2019	2	1.6	579.3	4,931,485	93,968	94	50.7	127	JAJNPB000000000	SAMN23498532	SRR17163594	10	Amp, Tet, Gen	*aph(6)-Id*, *bla*_TEM-1B_, *mef*(B), *strA*, *sul3*, *tet*(A)
CSCIP4_JAN2020	2	1.4	533.0	4,859,486	129,780	88	50.7	77	JAJNPC000000000	SAMN23498533	SRR17171575	69	Amp, Cip, Tmp, Chl	*bla*_TEM-1A_, *dfrA14*, *floR*, *tet*(A)
CSCIP2_JAN2020	2	1.5	541.9	4,861,961	106,396	89	50.8	114	JAJNPD000000000	SAMN23498534	SRR17171574	10	Amp, Cip, Tmp, Kan	*aph(3″)-Ib*, *aph(3′)-Ia*, *aph(6)-Id*, *bla*_TEM-1B_, *dfrA14*, *dfrA5*, *sul2*
CSCHL2_JAN2020	2	1.4	523.2	5,020,828	135,697	83	50.5	93	JAJNPE000000000	SAMN23498535	SRR17171573	127	Amp, Tet, Chl, Tmp, Gen	*aac(3)-IId*, *aadA5*, *aph(3″)-Ib*, *aph(6)-Id*, *bla*_TEM-1B_, *dfrA17*, *sul1*, *sul2*, *tet*(D)
CSCHL1_MAR2020	2	1.6	569.7	5,085,255	131,002	90	50.6	110	JAJNPF000000000	SAMN23498536	SRR17171572	131	Amp, Tet, Chl, Tmp, Gen	*aph(3″)-Ib*, *aph(6)-Id*, *bla*_TEM-1B_, *catA1*, *dfrA17*, *sul2*, *tet*(B)
CSCIP1_MAR2020	2	1.7	608.9	5,015,760	151,082	97	50.5	91	JAJNPG000000000	SAMN23498537	SRR17173283	457	Amp, Tet, Chl, Tmp, Kan, Gen, Ctx, Caz, Cip	*aac(3)-IId*, *aadA1*, *aph(3′)-Ia*, *bla*_CMY-2_, *bla*_TEM-1B_, *cmlA1*, *dfrA12*, *floR*, *lnu*(F), *sul2*, *sul3*, *tet*(A)
CSCIP2_MAR2020	2	1.7	612.3	5,367,993	93,535	91	50.6	120	JAJNPH000000000	SAMN23498538	SRR17173282	405	Amp, Tet, Cip, Ctx, Caz	*aac(3)-IId*, *aadA5*, *aph(3″)-Ib*, *aph(6)-Id*, *bla*_CTX-M-3_, *bla*_TEM-1B_, *dfrA17*, *mph*(A), *sul1*, *sul2*, *tet*(A)
SAMP1_JAN2019	3	1.6	602.7	5,134,503	130,024	94	50.6	115	JAJNPI000000000	SAMN23498539	SRR17173288	69	Amp, Tet	*bla*_TEM-1C_, *tet*(A)
SLAMP2_JAN2019	3	1.7	629.2	5,305,482	129,757	95	50.6	108	JAJNPJ000000000	SAMN23498540	SRR17173287	69	Amp, Tet	*bla*_TEM-1B_, *tet*(A)
GRCHL1_MAR2020	4	1.6	602.4	4,649,553	92,321	104	50.8	133	JAJNPK000000000	SAMN23498541	SRR17173286	58	Amp, Tet, Chl	*aph(3″)-Ib*, *aph(6)-Id*, *bla*_TEM-1B_, floR, *sul2*
GRCHL2_MAR2020	4	1.6	144.9	4,769,285	72,179	97	50.8	116	JAJNPL000000000	SAMN23498542	SRR17173285	216	Tet, Chl, Tmp	*aadA1*, *aadA2*, *aadA5*, *cmlA1*, *dfrA17*, *sul2*, *sul3*, *tet*(A)

a Sampling locations were as follows: 1, 43°16′49.5″S, 172°43′14.9″E; 2, 43°20′34.9″S, 172°38′14.1″E; 3, 43°23′27.7″S, 172°37′34.9″E; 4, 43°17′49.9″S, 172°39′04.7″E.

b AMR phenotype indicates resistance to antibiotics with MIC values at or above the CLSI breakpoint concentration. Amp, ampicillin (32 μg mL^−1^); Tet, tetracycline (16 μg mL^−1^); Chl, chloramphenicol (32 μg mL^−1^); Tmp, trimethoprim (16 μg mL^−1^); Kan, kanamycin (64 μg mL^−1^); Gen, gentamicin (16 μg mL^−1^); Ctx, cefotaxime (4 μg mL^−1^); Caz, ceftazidime (16 μg mL^−1^); Cip, ciprofloxacin (1 μg mL^−1^).

Resistance to sulfonamides, tetracycline, aminoglycosides, and β-lactam antibiotics represented the most common genotypes. Many of the ARGs have not been reported in the New Zealand environment, including *bla*_CTX-M-3_, *floR*, *cmlA1*, *inu*(F), *dfrA14*, *drfA5*, *mph*(A), *aadA1*, *aadA2*, *tet*(D), and *mef*(B) (Table 1).

We predicted that some of the ARGs were plasmid linked, and this was confirmed by the ability of seven strains to transmit one or more drug resistance phenotypes by conjugation (data not shown). The 15 draft genomes represent 11 sequence types (STs), including ST131 and ST457, known from community infections and animal reservoirs (18, 19) (Table 1).

We have reported draft genome sequences of AMR bacteria isolated from aquatic kai (foods) in Aotearoa, New Zealand. These data serve as a “wake-up call” regarding the risk of improper handling of aquatic wild foods and demonstrate the potential for their use as highly sensitive environmental monitors of AMR.

### Data availability.

The data have been deposited in GenBank under BioProject PRJNA784635, and the accession numbers are presented in Table 1.
